# Appropriate 'housekeeping' genes for use in expression profiling the effects of environmental estrogens in fish

**DOI:** 10.1186/1471-2199-8-10

**Published:** 2007-02-08

**Authors:** Amy L Filby, Charles R Tyler

**Affiliations:** 1 Environmental and Molecular Fish Biology Group, School of Biosciences, Hatherly Laboratories, University of Exeter, Prince of Wales Road, Exeter, Devon, EX4 4PS, UK

## Abstract

**Background:**

Attempts to develop a mechanistic understanding of the effects of environmental estrogens on fish are increasingly conducted at the level of gene expression. Appropriate application of real-time PCR in such studies requires the use of a stably expressed 'housekeeping' gene as an internal control to normalize for differences in the amount of starting template between samples.

**Results:**

We sought to identify appropriate genes for use as internal controls in experimental treatments with estrogen by analyzing the expression of eight functionally distinct 'housekeeping' genes (18S ribosomal RNA [18S rRNA], *ribosomal protein l8 *[*rpl8*], *elongation factor 1 alpha *[*ef1a*], *glucose-6-phosphate dehydrogenase *[*g6pd*], *beta actin *[*bactin*], *glyceraldehyde-3-phosphate dehydrogenase *[*gapdh*], *hypoxanthine phosphoribosyltransferase 1 *[*hprt1*], and *tata box binding protein *[*tbp*]) following exposure to the environmental estrogen, 17α-ethinylestradiol (EE_2_), in the fathead minnow (*Pimephales promelas*). Exposure to 10 ng/L EE_2 _for 21 days down-regulated the expression of *ef1a*, *g6pd*, *bactin *and *gapdh *in the liver, and *bactin *and *gapdh *in the gonad. Some of these effects were gender-specific, with *bactin *in the liver and *gapdh *in the gonad down-regulated by EE_2 _in males only. Furthermore, when *ef1a*, *g6pd*, *bactin *or *gapdh *were used for normalization, the hepatic expression of two genes of interest, *vitellogenin *(*vtg*) and *cytochrome P450 1A *(*cyp1a*) following exposure to EE_2 _was overestimated.

**Conclusion:**

Based on the data presented, we recommend 18S rRNA, *rpl8*, *hprt1 *and/or *tbp*, but not *ef1a*, *g6pd*, *bactin *and/or *gapdh*, as likely appropriate internal controls in real-time PCR studies of estrogens effects in fish. Our studies show that pre-validation of control genes considering the scope and nature of the experiments to be performed, including both gender and tissue type, is critical for accurate assessments of the effects of environmental estrogens on gene expression in fish.

## Background

Over the past 25 years, there has been increasing concern about the impacts of the plethora of natural and anthropogenic chemicals discharged into the aquatic environment that disrupt the endocrine system of wildlife [1]. Typically, these endocrine disrupting chemicals (EDCs) exert their actions via interactions with the nuclear steroid hormone receptors. The most well characterised, and of greatest current concern, are EDCs which bind to and activate the estrogen receptor: so-called environmental estrogens (for a review, see [2]). The international research effort on chemically-induced endocrine disruption has been driven by unequivocal evidence that environmental estrogens alter sexual development and function in fish (for a review, see [3]), and effects on other physiological processes, including growth, development, osmoregulation, and stress and immune responses, have also been reported (reviewed in [4]). An improved understanding of the mechanisms by which these effects occur is critical if we are to fully assess the potential health implications of exposure to environmental estrogens, both for fish and for other vertebrates.

Attempts to develop a mechanistic understanding of the effects of environmental estrogens in the body are increasingly being conducted at the level of gene expression (reviewed in [5]). Real-time reverse transcription PCR is presently the most sensitive method for the detection of mRNAs [6], and is also often employed for the validation of gene expression data from high-throughput array experiments [7]. Quantitative analysis of gene expression using real-time PCR typically requires the use of a constitutively expressed 'housekeeping' gene as an internal control to normalize for differences in starting cDNA template between samples (reviewed in [8,9]). The fundamental requirement for validation of the expression stability of an internal control gene prior to its use in the system being studied is also well-defined. Nevertheless, in contrast to the situation for many mammalian experimental systems (e.g. [10-12]), studies investigating the effects of environmental estrogens on gene expression in non-mammalian vertebrates have used 'housekeeping' genes more or less randomly as internal controls, and without any validation of their expression stability in the system being studied, which may have serious implications for the interpretation of the effects data.

This study, therefore, set out to assess different 'housekeeping' genes for their potential use as internal controls to normalize for expression of genes of interest in experimental treatments with estrogen in fish. Eight 'housekeeping' genes were selected for assessment, including 18S ribosomal RNA (18S rRNA), *ribosomal protein l8 *(*rpl8*), *elongation factor 1 alpha *(*ef1a*), *glucose-6-phosphate dehydrogenase *(*g6pd*), *beta actin *(*bactin*), *glyceraldehyde-3-phosphate dehydrogenase *(*gapdh*), *hypoxanthine phosphoribosyltransferase 1 *(*hprt1*), and *tata box binding protein *(*tbp*) in the fathead minnow (*Pimephales promelas*; a fish species widely used in ecotoxicology). These genes were chosen based on their previous use as internal controls in gene expression studies, the availability of 'housekeeping' gene sequences in fathead minnow and related teleost species, and because they have roles in different cellular functions (ribosomal structure [18S rRNA], protein biosynthesis [*rpl8 *and *ef1a*], cytoskeletal structure [*bactin*], glucose metabolism [*g6pd *and *gapdh*], nucleoside metabolism [*hprt1*]), and transcription initiation [*tbp*]) thus reducing the likelihood that they exhibited regulated covariation. The genes of interest chosen to quantify responses to estrogen were *vitellogenin *(*vtg*), which encodes a protein that acts as a precursor of yolk, and *cytochrome P450 1A *(*cyp1a*), that codes for an enzyme involved in the metabolism of xenobiotics, both of which are frequently used for studies on environmental estrogens in fish.

Our results showed that the expression of some 'housekeeping' genes typically used for studies on environmental estrogens is altered by exposure to the environmental estrogen 17α-ethinylestradiol (EE_2_) and that control gene validation prior to experimental work is critical to the interpretation of environmental estrogen-related gene expression profiles derived using real-time PCR techniques. Our findings on the use of control genes for estrogen exposure studies in fish are likely to apply to other experimental/chemical manipulations.

## Results

### General expression levels of candidate 'housekeeping' genes

We first (based on mean real-time PCR threshold cycle [C_t_] values in control fish) calculated the general expression levels of the candidate 'housekeeping' genes, since extremely high or low expression levels may preclude the usefulness of these genes as internal controls (Table 1). 18S rRNA was by far the most highly expressed gene in both tissues studied. *ef1a *and *rpl8 *were also consistently among the most highly expressed 'housekeeping' genes, while *tbp*, *hprt1 *and *g6pd *were consistently among the lowest expressed genes. *gapdh *was highly expressed in liver, but among the lowest expressed 'housekeeping' genes in gonad, while the opposite was true for *bactin*, which was highly expressed in gonad but had a low level of expression in liver.

### Expression of candidate 'housekeeping' genes following exposure to estrogen

**Figure 1:**
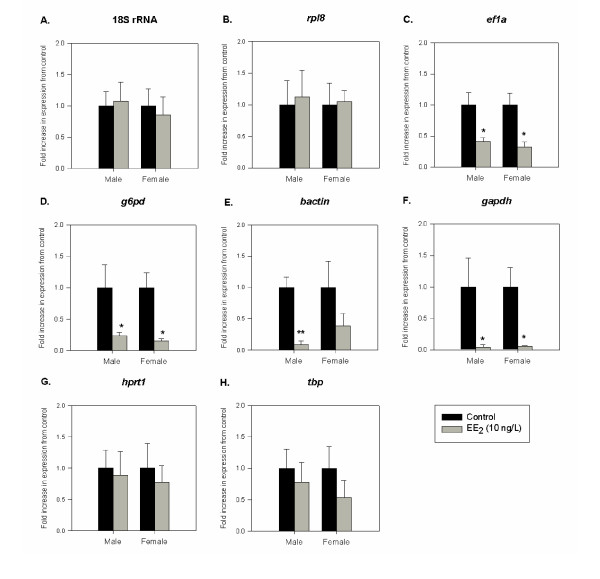
**Expression of the candidate 'housekeeping' genes in fathead minnow liver following exposure to 10 ng EE_2_/L**. (A) 18S rRNA, (B) *rpl8*, (C) *ef1a*, (D) *g6pd*, (E) *bactin*, (F) *gapdh*, (G) *hprt1*, and (H) *tbp*. The results are represented as means ± S.E.M. and expressed as fold-increase in expression from the control which was arbitrarily set as the 1× expression of the gene. Each treatment group consisted of eight male and eight female fish and each fish was analysed in triplicate. Statistically significant differences in gene expression between control and EE_2_-treated fish for each sex are denoted as follows: * *P *< 0.05, ** *P *< 0.01, *** *P *< 0.001 (Student's *t*-test).

The expression levels of some of the candidate 'housekeeping' genes were altered in adult fathead minnow following 21 days exposure to EE_2_, but the effects of EE_2 _in some cases differed depending on the tissue type (liver or gonad) and/or the gender of the fish. In liver (Fig. 1), there was no effect of this exposure on the expression of 18S rRNA, *rpl8*, *hprt1 *or *tbp *in either males or females (Fig. 1A,B,G, and 1H). However, expression of *ef1a*, *g6pd*, and *gapdh *in liver was down-regulated following exposure to EE_2 _in both males and females (*ef1a*: to 32–41% of the control level; *g6pd*: to 15–23% of the control level; *gapdh*: to 4–6% of the control level) (Figs. 1C,D, and 1F). Expression of *bactin *in liver was also down-regulated following exposure to EE_2 _but only significantly so in males (to 9% of the control level) (Fig. 1E).

**Figure 2:**
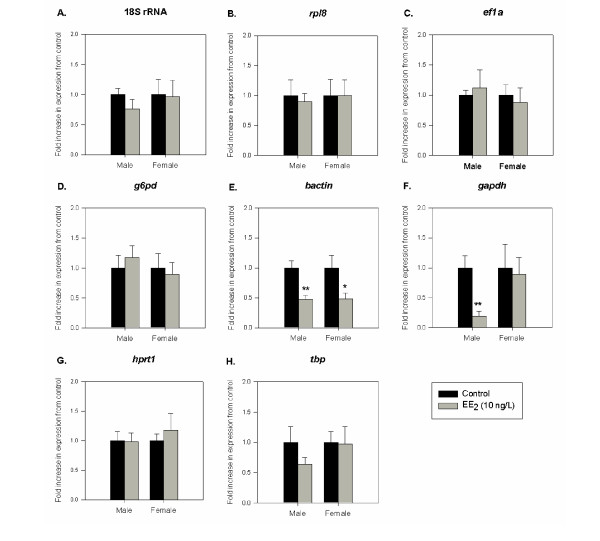
**Expression of the candidate 'housekeeping' genes in fathead minnow gonad following exposure to 10 ng EE_2_/L**. (A) 18S rRNA, (B) *rpl8*, (C) *ef1a*, (D) *g6pd*, (E) *bactin*, (F) *gapdh*, (G) *hprt1*, and (H) *tbp*. The results are represented as means ± S.E.M. and expressed as fold-increase in expression from the control which was arbitrarily set as the 1× expression of the gene. Each treatment group consisted of eight male and eight female fish and each fish was analysed in triplicate. Statistically significant differences in gene expression between control and EE_2_-treated fish for each sex are denoted as follows: * *P *< 0.05, ** *P *< 0.01, *** *P *< 0.001 (Student's *t*-test).

In gonad (Fig. 2), the expression levels of 18S rRNA, *rpl8*, *ef1a*, *g6pd*, *hprt1 *and *tbp *were unaffected following exposure to EE_2 _in both males and females. However, the expression levels of *bactin *(in both males and females) and *gapdh *(in males only) were reduced following exposure to EE_2 _(for *bactin*, to 47–48% of the control level, Fig. 2E; for *gapdh*, to 19% of the control level, Fig. 2F).

### The effect of using the different 'housekeeping' genes to normalize data for genes of interest

**Figure 3:**
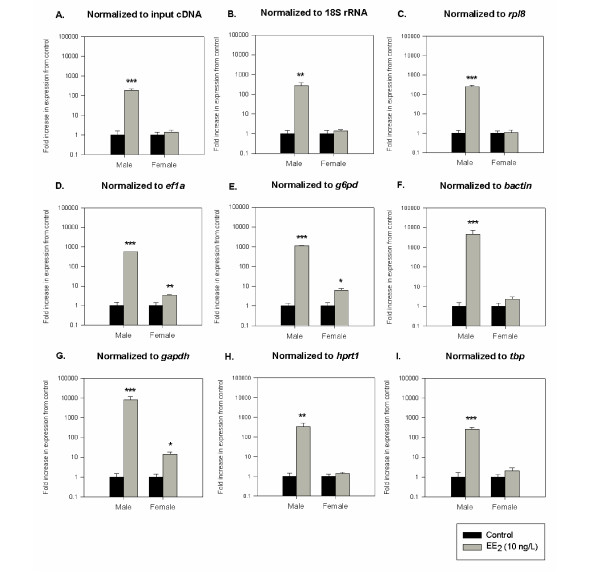
**Expression of the gene of interest *vtg *in fathead minnow liver following exposure to 10 ng EE_2_/L**. *vtg *expression data is presented (A) normalized to input cDNA, or normalized to the expression of different 'housekeeping' genes: (B) 18S rRNA, (C) *rpl8*, (D) *ef1a*, (E) *g6pd*, (F) *bactin*, (G) *gapdh*, (H) *hprt1*, and (I) *tbp*, measured in each sample. The results are represented as means ± S.E.M., expressed as fold-increase in expression from the control which was arbitrarily set as the 1× expression of the gene, and shown on a logarithmic scale. Each treatment group consisted of eight male and eight female fish and each fish was analysed in triplicate. Statistically significant differences in gene expression between control and EE_2_-treated fish for each sex are denoted as follows: * *P *< 0.05, ** *P *< 0.01, *** *P *< 0.001 (Student's *t*-test).

When the expression data were normalized to the amount of input cDNA, *vtg *was, as expected, shown to be substantially (190-fold) up-regulated in liver of male fathead minnow exposed to EE_2 _(Fig. 3A) but not in females. *vtg *expression in male liver (but not in female liver) was also shown to be significantly increased by EE_2 _exposure, and by a similar fold, when the data were instead normalized using 'housekeeping' genes whose expression were unchanged by estrogen in this tissue (247-340-fold; Figs. 3B,C,H and 3I). Although *vtg *expression in male liver was also shown to be up-regulated by EE_2 _when *ef1a*, *g6pd*, *bactin *or *gapdh *('housekeeping' genes down-regulated by EE_2 _in this tissue) were used for the normalization (Figs. 3D–G), the fold-increases in *vtg *expression compared to control males using these 'housekeeping' genes were 563 ± 19-fold for *ef1a*, 1090 ± 75-fold for *g6pd*, 4515 ± 2758-fold for *bactin*, and 8341 ± 3211-fold for *gapdh*, which were significantly higher values compared with those obtained using the 'housekeeping' genes not affected by the EE_2 _treatment. A further difference observed when normalizing to *ef1a*, *g6pd *or *gapdh *was that *vtg *expression in female liver was over-estimated and shown to be increased significantly with EE_2 _treatment (for *ef1a*, by 3.4-fold, for *g6pd*, by 6.34-fold, and for *gapdh*, by 15-fold).

**Figure 4:**
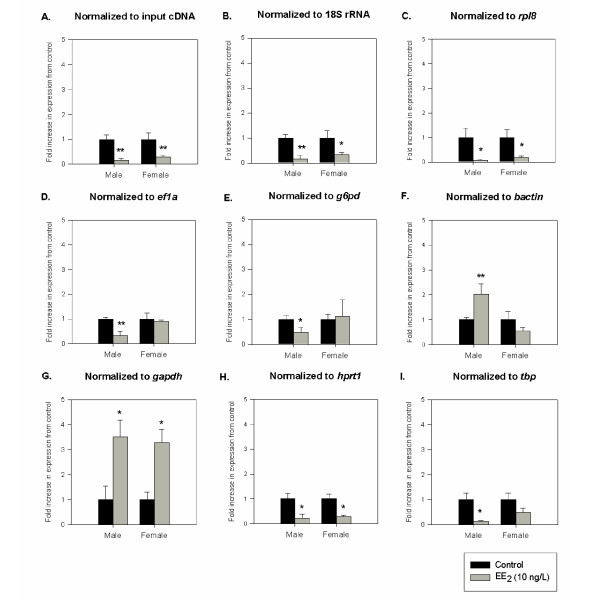
**Expression of the gene of interest *cyp1a *in fathead minnow liver following exposure to 10 ng EE_2_/L**. *cyp1a *expression data is presented (A) normalized to input cDNA, or normalized to the expression of different 'housekeeping' genes: (B) 18S rRNA, (C) *rpl8*, (D) *ef1a*, (E) *g6pd*, (F) *bactin*, (G) *gapdh*, (H) *hprt1*, and (I) *tbp*, measured in each sample. The results are represented as means ± S.E.M., expressed as fold-increase in expression from the control which was arbitrarily set as the 1× expression of the gene. Each treatment group consisted of eight male and eight female fish and each fish was analysed in triplicate. Statistically significant differences in gene expression between control and EE_2_-treated fish for each sex are denoted as follows: * *P *< 0.05, ** *P *< 0.01, *** *P *< 0.001 (Student's *t*-test).

When the expression data were normalized to the amount of input cDNA, *cyp1a *was, as expected, shown to be down-regulated in liver of fathead minnow exposed to EE_2 _in both males and females (to 15–28% of the control level; Fig. 4A). Similarly, *cyp1a *expression in liver was also shown to be significantly decreased by EE_2 _when *cyp1a *expression was instead normalized to the expression of either 18S rRNA, *rpl8*, or *hprt1 *in both males (18S rRNA: to 17% of the control level;*rpl8*: to 8% of the control level; *hprt1*: to 22% of the control level) and females (18S rRNA: to 34% of the control level; *rpl8*: to 19% of the control level; *hprt1*: to 29% of the control level) (Figs. 4B,C,H) and *tbp *in males (*tbp*: to 14% of the control level) (Fig. 4I). *cyp1a *expression was also reduced in males exposed to EE_2 _when *ef1a *and *g6pd *were used for the normalizations, but to a smaller extent (*ef1a*: to 34% of the control level; *g6pd*: to 49% of the control level). (Figs. 4D–E). In contrast, in females EE_2 _had no significant effect on *cyp1a *expression when *ef1a *or *g6pd *was used for the normalization. Moreover, when *bactin *and *gapdh *were used for the normalization, *cyp1a *expression apparently increased with EE_2 _exposure in males (*bactin*; 2-fold) or in both males and females (*gapdh*; males: 3.5-fold, females: 3.3-fold) (Figs. 4F–G).

## Discussion

In this study, we investigated a suite of eight 'housekeeping' genes that represent different functional classes and gene families for use as internal controls to normalize real-time PCR data in experimental treatments with an estrogen (EE_2_) in fish. Specifically, these genes comprised 18S rRNA, *rpl8*, *ef1a*, *g6pd*, *bactin*, *gapdh*, *hprt1*, and *tbp*. These 'housekeeping' genes have been validated previously for use as internal controls in many experimental systems, particularly in mammals (18S rRNA: e.g. [10-12]; ribosomal proteins: e.g. [13-15]; *ef1a*: e.g. [13,14,16]; *g6pd*: e.g. [17,18]; *bactin*: e.g. [10,19]), *gapdh*: e.g. [19,20]; *hprt1*: e.g. [19,21-23]; *tbp *e.g. [19]), but have not been validated for work on estrogens in fish. The development of the required real-time PCR assays for some of the target 'housekeeping' genes, whose sequences were not publicly available in the study species (18S rRNA, *rp18*, *ef1a*), first required the cloning of the full- or partial-length sequences for these genes.

A wide range of 'housekeeping' genes have been used as internal controls in expression profiling following exposure of fish to estrogens, including *bactin *(e.g. 17β-estradiol [E_2_] [24,25]; EE_2 _[26-28]; bisphenol A [BPA] [26]; nonylphenol [NP] [26]; alpha-zearalenol [24]), 16S rRNA (e.g. EE_2 _[29]), 18S rRNA (e.g. E_2 _[30,31]; NP [30]), *ef1a *(e.g. EE_2 _[32]; NP [32]), and ribosomal proteins (including S2, S3, S8, S15, S27, L4, L5, L8, L13, L21, and L28; e.g. E_2_, NP, and 1,1-dichloro-2 2-bis (*p*-chlorophenyl) ethylene [*p*,*p'*-DDE]: [33]). However, very few of those studies provided any validation of these genes for use as internal controls prior to their application.

Our data clearly show that the expression levels of some 'housekeeping' genes are regulated by estrogen in fathead minnow. In particular, *ef1a*, *g6pd*, *bactin*, and *gapdh *were found to be strongly regulated by estrogen, and this is consistent with data published for other teleosts. Down-regulation of *bactin *was shown to occur in array analyses conducted on liver tissue from male sheepshead minnow (*Cyprinodon variegates*) exposed to the environmental estrogens E_2_, EE_2_, diethylstilbestrol (DES), NP, and methoxychlor [5]. This concurs with the observations that E_2 _and several other estrogenic compounds disrupt cytoskeletal compounds in vitro [34-36]. In contrast, *bactin *expression has been shown to be increased in liver of plaice (*Pleuronectes platessa*) and zebrafish (*Danio rerio*) exposed to EE_2 _[29,37], and in pituitary of Atlantic salmon (*Salmo salar*) exposed to E_2 _and NP [38]. Expression of *gapdh *in livers of plaice and rainbow trout (*Oncorhynchus mykiss*) has also been shown to be repressed by various environmental estrogens, including EE_2 _[28,29,39]. Further evidence of estrogen-regulation of *bactin *and *gapdh *coming from mammalian studies [40-43] strengthens the case that their use as internal controls for studies for quantification of gene expression with estrogen treatment is probably inappropriate. The down-regulation of hepatic *ef1a *and *g6pd *by EE_2 _observed in the present study also concurs with data from zebrafish embryos [39], where two *ef1a *isoforms were down-regulated by estrogen, and from the European flounder (*Platichthys flesus*), where E_2 _inhibited activity of the G6pd enzyme in hepatocytes by 30% in males and 80% in females [44]. In fact, estrogen-regulation of *g6pd *provides a likely explanation for differences we observed in hepatic *g6pd *expression between the sexes in this study (lower expression in females; data not shown), since female fish have higher circulating levels of endogenous estrogens than males. The effect of estrogen on *gapdh *and *g6pd *expression observed here likely results from their involvement in metabolism, since estrogens, like other sex steroids, are well known to have roles in the regulation of the metabolic processes associated with altered energy demands during gonad development and reproduction in fish [45-47]. Moreover, several other metabolic enzymes are also known to be controlled by sex steroids including estrogens in fish [27,46,48].

Interestingly, for some of the candidate 'housekeeping' genes, the effects of estrogen were tissue- and/or gender-specific. For example, *ef1a *and *g6pd *expression were only altered by EE_2 _in liver and not in gonad. Moreover, the expression levels of *gapdh *in gonad and *bactin *in liver were only down-regulated by EE_2 _in males and not in females. Complex effects of estrogens (including tissue-, gender-, and/or developmental-stage-specific effects) have been demonstrated for many estrogen-regulated genes, including 'housekeeping' genes, in fish and higher vertebrates (e.g. [4,41]) and this suggests that these genes may have different roles in different tissues, and different roles in males compared with in females in these tissues. The tissue- and gender-specific nature of these effects of EE_2 _on 'housekeeping' gene expression emphasises the need for caution if extrapolating data from one tissue/gender to another when choosing an internal control gene.

Four of the candidate 'housekeeping' genes (18S rRNA, *rpl8*, *hprt1 *and *tbp*) were found to be unaffected by estrogen treatment in both tissues examined in this work and are, therefore, considered valid for use as internal controls in such experiments. The high stability of *rpl8 *expression following estrogen treatment conforms with data from largemouth bass (*Micropterus salmoides*), where the expression of a suite of 11 ribosomal proteins, including *rpl8*, did not fluctuate appreciably (<1.3-fold) in response to estrogenic compounds, and ribosomal proteins were thus recommended for use as internal control genes in microarray experiments involving estrogen treatments [33]. However, the results of another study on rainbow trout demonstrated EE_2_-induction of the ribosomal protein *ribosomal protein s3 *(*rps3*) [28], emphasising that not all ribosomal proteins may be estrogen-independent. Although there is no other data currently available concerning possible estrogen regulation of *hprt1 *and *tbp *in fish, *hprt *has been described as an estrogen-independent 'housekeeping' gene in mammals [21], supporting our findings here.

The lack of regulation of 18S rRNA by EE_2 _in this study (21 day exposure) concurs with the findings for an exposure to EE_2 _conducted on zebrafish (liver, for periods of 48 and 168 hours) [37]. In that study however, and based on array analyses, 18S rRNA was up-regulated in a concentration-dependent manner in zebrafish liver after a shorter term (24 hour) exposure to EE_2_, suggesting that any 18S rRNA regulation by estrogen may occur in a temporal manner. Although rRNAs have been advocated for use as internal controls in many other experimental systems and their levels are thought to be less likely to vary under conditions that affect the expression of mRNAs (e.g. [10-12,49]), there is some evidence that another rRNA, 28S rRNA, is also regulated by estrogen in fish [29]. Moreover, some clear disadvantages in the use of rRNAs as internal controls have been highlighted by other researchers (e.g. see [17,50,51]. Most notably, rRNAs can only be used as internal controls for total RNA preparations (since mRNA purification methods randomly remove them) and cannot be used when an oligo(dT) reverse transcription has been carried out. The use of rRNAs, which constitute 80–90% of total RNA, has also been criticised due to their very high general expression level which far exceeds that for mRNAs (e.g. [51,52]). A very high general level of expression of 18S rRNA was also observed in the current study (18S rRNA had a mean C_t _of approximately 11, compared to mean C_t_s of approximately 23–32 for the other candidate 'housekeeping' genes). It is imperative, therefore, that these issues are considered if selecting 18S rRNA as an internal control for studies on environmental estrogens in fish.

To assess the importance of selecting an appropriately validated 'housekeeping' gene for use as an internal control, in our final analyses we measured the expression of two genes of interest, *vtg *and *cyp1a*, in the livers of fathead minnow exposed to EE_2 _and compared the relative expression results obtained using the different housekeeping genes to those obtained when the amount of input cDNA was normalized. *vtg *and *cyp1a *were selected for this analysis because of their biological roles in oocyte development and xenobiotic metabolism, respectively, because they are well characterised as estrogen-inducible and estrogen-downregulated genes, respectively, and because they are frequently included as genes of interest in studies on the effects of environmental estrogens (e.g. [32,53,54]). When the amount of input cDNA was normalized, *vtg *mRNA expression in liver was highly (approximately 200-fold) up-regulated by EE_2 _in males (but not in females), while *cyp1a *mRNA expression was down-regulated by EE_2 _in both sexes (to between 15–28% of the control level), and these findings were as expected and in agreement with the current literature (e.g. [32,53,54]). We then alternatively normalized *vtg *and *cyp1a *expression using a 'housekeeping' gene approach, with each of the eight different 'housekeeping' genes.

Depending on the 'housekeeping' gene used for the gene of interest normalization, *vtg *mRNA levels were either unchanged or increased (to widely varying degrees from 250- to 8,300-fold), and *cyp1a *mRNA levels were either down-regulated, unchanged or increased (and, again, to different degrees). As expected, the use of appropriate 'housekeeping' genes (18S rRNA, *rpl8, tbp*, or *hprt1*; which were not affected by the EE_2 _treatment) for the normalizations, gave very similar results to the data generated when the input cDNA amount was normalized, demonstrating that the use of these genes is valid and effectively normalizes for differences in input cDNA. In contrast, when 'housekeeping' genes down-regulated by the EE_2 _treatment (*ef1a*, *g6pd*, *bactin*, or *gapdh*) were used, the expression levels of the genes of interest were overestimated, which lead to inappropriate conclusions about the manner in which these genes were regulated, or not regulated, by estrogen, and/or the degree to which they were estrogen-regulated. Likewise, if a 'housekeeping' gene which was up-regulated by EE_2 _had been used for normalization, the expression levels of the genes of interest would have been underestimated. These findings have major implications for studies on the effects and potential impacts of environmental estrogens in fish that use internal control genes that have not been validated for the specifics of the experimental system, and in particular for studies which require to detect small changes in the expression of the gene of interest. An interesting aside from this work has been to show that an environmental concentration of EE_2 _is capable of disrupting expression of a suite of genes that play key roles in general cell function and maintenance, adding further to the concern about the potential impacts of this estrogen in the aquatic environment.

## Conclusion

Our results have shown that, in fish, the expression levels of some of the most commonly used 'housekeeping' genes are, in fact, regulated by estrogen treatment, and based on the data presented, we therefore recommend against the use of *ef1a*, *g6pd*, *bactin*, and *gapdh *as internal controls in real-time PCR studies of estrogen-exposed fish. In contrast, based on their estrogen-independency in this study, we recommend 18S rRNA, *rpl8*, *hprt1 *and *tbp *as likely appropriate internal control genes in this experimental setting. Given that these data have shown that choosing an inappropriate 'housekeeping' gene as an internal control to normalize expression data for a gene of interest can potentially lead to inaccurate conclusions on estrogen effect pathways, we strongly recommend that most appropriate 'housekeeping' gene for use as an internal control is first established for the specifics of each estrogen experiment. This selection should take into account the fact that estrogen-regulation of 'housekeeping' genes may vary with factors such as chemical, dose and, exposure length, in addition to gender and tissue type.

## Methods

### Estrogen exposure

The synthetic estrogen EE_2_, a contaminant widespread in the aquatic environment (e.g. [55]), was chosen to determine the effects of estrogen treatment on expression of the candidate 'housekeeping' genes studied. Eight tanks of adult (>150 days post hatch) male and female fathead minnow (1 male and 1 female per tank) were exposed to 10 ng EE_2_/L (Sigma, Poole, U.K.) under flow-through conditions for a period of 21 days. Eight tanks with the same numbers of fish were maintained in dilution water as controls. The test concentration adopted in this work was within the concentration range found in European wastewater treatment works (WwTW) effluents (e.g. [55]). Fish were sacrificed by a lethal overdose of anesthesia (500 mg/L MS-222 [3-aminobenzoic acid ethyl ester] buffered to pH 7.4; Sigma). Liver and gonad tissue samples were collected from each fish from a single replicate tank, snap-frozen in liquid nitrogen and stored at -80°C until RNA extraction for subsequent gene expression analyses.

### RNA extraction and cDNA synthesis

Total RNA was extracted from the tissue samples using Tri Reagent (Sigma), following manufacturer's instructions. Total RNA concentration was calculated from absorbance at 260 nm (A_260nm_; GeneQuant; Amersham Biosciences, Little Chalfont, U.K.) and RNA quality was verified by electrophoresis on ethidium bromide-stained 1.5% agarose gels and by A_260nm_/A_280nm _ratios >1.8. For each sample, 2 μg total RNA was treated with RQ1 DNase (Promega, Southampton, U.K.) to remove any contaminating DNA. First strand cDNA was synthesized from the DNase-treated RNA using random hexamer primers (5'-NNNNNN-3'; MWG-Biotech, Ebersburg, Germany) and Moloney-murine leukemia virus (M-MLV) reverse transcriptase (Promega), following manufacturer's instructions.

### cDNA quantification and normalization

To remove the RNA strand (which would interfere with cDNA quantification), the cDNA was treated with RNase by adding 1 μl RNase Cocktail (Ambion, Huntingdon, U.K.) per 25 μl cDNA and incubating for 30 min at 37°C followed by 10 min at 70°C to deactivate the enzyme. The cDNA was purified using NucAway Spin Columns (Ambion) to remove chemical contaminants and free nucleotides and then quantified in duplicate with the Quant-iT OliGreen ssDNA reagent kit (Invitrogen) according to the manufacturer's instructions using a Tecan GENios FL fluorescence microplate reader (Tecan U.K. Ltd., Theale, U.K.). No-RNase controls (to which the RNase Cocktail was substituted for molecular-grade water during the RNase treatment step) were also quantified using OliGreen to ensure that the RNase step had worked. An aliquot of each cDNA sample was then normalized (by dilution with molecular-grade water) such that all the samples contained equal amounts (20 ng/μl) of cDNA template for real-time PCR.

### Development of real-time PCR assays for candidate 'housekeeping' genes

#### Cloning of cDNAs for candidate 'housekeeping' genes

For three of the target 'housekeeping' genes (18S rRNA, *rpl8 *and *ef1a*), there was no cDNA sequence information available for the fathead minnow and, therefore, their cDNA sequences had to first be cloned and sequenced to enable the design of primers for real-time PCR amplification of their transcripts. Full-length (for *ef1a*) or partial-length (for 18S rRNA and *rpl8*) sequences were obtained from fathead minnow whole body tissue using reverse transcription PCR (RT-PCR), 5'-/3'-Rapid Amplification of cDNA Ends (RACE), cloning, and automated fluorescence sequencing strategies as described in detail previously [56]. The full-length fathead minnow *ef1a *cDNA sequence [1748 bp; GenBank: AY643400] was obtained in four consecutive steps consisting of (a) an RT-PCR to obtain a core partial-length fragment of the cDNA using primers designed from regions of *ef1a *cDNAs conserved between fish species available in the NCBI GenBank database ('Core PCR 1'), (b) a second RT-PCR to extend the initial core partial-length fragment towards the 5'-end ('Core PCR 2'), (c) a 5'-RACE to obtain the 5'-end of the cDNA ('5'-RACE'), and (d) a 3'-RACE to obtain the 3'-end of the cDNA ('3'-RACE'). The partial-length fathead minnow 18S rRNA (768 bp; GenBank: AY855349) and *rpl8 *(708 bp; GenBank: AY919670) sequences were obtained by separate RT-PCRs using primers designed from conserved regions of 18S rRNAs and *rpl8 *cDNAs available in the NCBI GenBank database. For PCR primer sequences, annealing temperatures, cycle numbers and product sizes, see Additional File 1: Filby and Tyler Additional File 1. Sequence identities were confirmed using BLASTn [57] and phylogenetic analyses within CLUSTAL X [58].

#### Development of real-time PCR assays

Primers specific for fathead minnow 18S rRNA, and for fathead minnow *rpl8*, *ef1a*, *g6pd *[GenBank: AF206637], *bactin *[GenBank: DT257898], *gapdh *[GenBank: DT353565], *hprt1 *[GenBank: DT085800], and *tbp *[GenBank: DT344258] cDNAs, were designed with Beacon Designer 3.0 software (Premier Biosoft International, Palo Alto, CA, USA) according to the manufacturer's guidelines and purchased from MWG-Biotech. Assays were optimized and validated for real-time quantitative PCR using SYBR Green chemistry as described previously [56]. All assays had detection ranges of at least five orders of magnitude. Specificity of primer sets throughout this range of detection was confirmed by the observation of single amplification products of the expected size, melting temperature and sequence. All assays were quantitative, with standard curve (mean C_t _vs. log cDNA dilution) slopes of between -2.794 and -3.533, translating to high real-time PCR efficiency (*E*) values of 1.92–2.28. Over the detection range, the linear correlation (R_2_) between the mean C_t _and the logarithm of the cDNA dilution was >0.99 in each case. For real-time PCR primer sequences, product sizes, annealing temperatures, and standard curve slope, PCR efficiency, and correlation coefficient values, see Additional File 2: Filby and Tyler Additional File 2.

### Real-time PCR to determine expression of candidate 'housekeeping' genes

Real-time PCR using SYBR Green chemistry was performed on 20 ng cDNA with the iCycler iQ Real-time Detection System (Bio-Rad Laboratories, Inc., Hercules, CA, U.S.A.) using the protocol described previously [56]. Assays had a high level of precision and reproducibility with intra-assay coefficient of variation of 2.42 %; (n = 96). Inter-assay coefficient of variation was not measured because all of the samples for each gene were run on the same plate. The expression of each 'housekeeping' gene was calculated using the 2^-ΔCt ^method described in [59] but employed the actual measured *E *value for each 'housekeeping' gene rather than assuming *E *= 2 ('efficiency correction'). The 2^-ΔCt ^formula is a modification of the arithmetic comparative 2^ΔΔCt ^method [59] that was developed to enable normalization to a measurement external to the PCR experiment (in this case, input cDNA), for measuring the expression of 'housekeeping' genes following different treatments. This method expressed the data as the fold-increase in expression from the mean of the experimental group set as the calibrator (the control treatment) and, therefore, enabled direct comparison of the expression of 'housekeeping' genes with different general levels of expression.

### Effect of using the different 'housekeeping' genes to normalize data for genes of interest

To determine the effect of using the different 'housekeeping' genes on normalized data for genes of interest, the liver cDNA samples from the EE_2 _exposure which had not been normalized to contain equal cDNA concentrations were analysed by real-time PCR (as described above) for the expression of the two estrogen-regulated genes of interest *vtg*, an estrogen-upregulated gene [32,53], and *cyp1a*, an estrogen-downregulated gene [54], in parallel with expression analyses of the eight candidate 'housekeeping' genes. The fathead minnow *vtg *[Genbank: AF130354] assay was as described previously [4]. The primers for the fathead minnow *cyp1a *[Genbank: AF232749] assay were 5'-AGGGAGAACTGAGAGAG-3'/5'-GTGAGGGATGGTGAAC-3' and the annealing temperature was 57°C. Relative expression levels (gene of interest: 'housekeeping' gene) were determined using a development of the arithmetic comparative 2^-ΔΔCt ^method [59] which includes efficiency correction [60,61].

### Statistical analyses

Comparison of means was carried out with the Student's *t*-test or One-Way Analysis of Variance followed by Tukey's post hoc test using SigmaStat 2.03 software (Jandel Scientific Software). Non-normally distributed data were log-transformed prior to statistical analysis and experimental data are shown as the mean ± S.E.M.

## Authors' contributions

ALF was responsible for the carrying out the experiments and data analyses and drafted the manuscript. CRT participated as a supervisor in study design and analyses and edited the manuscript. Both authors read and approved the final manuscript.

## Supplementary Material

Additional file 1 Table of PCR primer sequences, annealing temperatures (T_a_), cycle numbers and product sizes used for cloning the candidate 'housekeeping' genes. Click here for file

Additional file 2 Table of real-time PCR primer sequences, real-time PCR product sizes, annealing temperatures (T_a_), and standard curve (mean C_t _vs. log cDNA dilution) slopes, PCR efficiencies (*E*), and correlation coefficients for the candidate 'housekeeping' genes. Click here for file

## Figures and Tables

**Table 1:** General expression levels of the candidate 'housekeeping' genes in fathead minnow liver and gonad tissues.

**LIVER**		**GONAD**	
**'Housekeeping' Gene**	**C_t _(Mean ± S.E.)**	**'Housekeeping' Gene**	**C_t _(Mean ± S.E.)**

18S rRNA	11.72 ± 0.35	18S rRNA	11.58 ± 0.32
*ef1a*	22.55 ± 0.18	*bactin*	23.24 ± 0.29
*gapdh*	23.95 ± 0.41	*ef1a*	23.34 ± 0.43
*rpl8*	25.65 ± 0.51	*rpl8*	24.78 ± 0.65
*bactin*	28.12 ± 0.64	*tbp*	25.74 ± 0.24
*g6pd*	28.40 ± 0.50	*gapdh*	27.26 ± 0.37
*hprt1*	29.51 ± 0.67	*hprt1*	28.68 ± 0.22
*tbp*	31.96 ± 0.56	*g6pd*	29.76 ± 0.24

Genes are shown from highest expressed (low C_t_, top) to lowest expressed (high C_t_, bottom) for each tissue based on mean real-time PCR C_t _values in control fish.

## References

[B1] Tyler CR, Jobling S, Sumpter JP (1998). Endocrine disruption in wildlife: a critical review of the evidence. Critical Reviews in Toxicology.

[B2] Sonnenschein C, Soto AM (1998). An updated review of environmental estrogen and androgen mimics and antagonists. Journal of Steroid Biochemistry and Molecular Biology.

[B3] Mills LJ, Chichester C (2005). Review of evidence: Are endocrine-disrupting chemicals in the aquatic environment impacting fish populations. The Science of The Total Environment.

[B4] Filby AL, Thorpe KL, Tyler CR (2006). Multiple molecular effect pathways for disruption of physiological function by an environmental oestrogen in fish. Journal of Molecular Endocrinology.

[B5] Larkin P, Folmar LC, Hemmer MJ, Poston AJ, Denslow ND (2003). Expression profiling of estrogenic compounds using a sheepshead minnow cDNA macroarray. EHP Toxicogenomics.

[B6] Bustin SA (2000). Absolute quantitation of mRNA using real-time reverse transcription polymerase chain reaction assays. Journal of Molecular Endocrinology.

[B7] Rajeevan MS, Vernon SD, Taysavang N, Unger ER (2001). Validation of array-based gene expression profiles by real-time (kinetic) RT-PCR. Journal of Molecular Diagnostics.

[B8] Sturzenbaum SR, Kille P (2001). Control genes in quantitative molecular biological techniques: the variability of invariance. Comparative Biochemistry and Physiology B.

[B9] Bustin SA (2002). Quantification of mRNA using real-time reverse transcription PCR (RT-PCR): trends and problems. Journal of Molecular Endocrinology.

[B10] Morse DL, Carroll D, Weberg L, Borgstrom MC, Ranger-Moore J, Gillies RJ (2005). Determining suitable internal standards for mRNA quantification of increasing cancer progression in human breast cells by real-time reverse transcriptase polymerase chain reaction. Analytical Biochemistry.

[B11] Goidin D, Mamessier A, Staquet MJ, Schmitt D, Berthier-Vergnes O (2001). Ribosomal 18S RNA prevails over gluceraldehyde-3-phosphate dehydrogenase and β-actin genes as internal standard for quantitative comparison of mRNA levels in invasive and noninvasive human melanoma cell subpopulations. Analytical Biochemistry.

[B12] Schmid H, Cohen CD, Henger A, Irrgang S, Schlondorff D, Kretzler M (2003). Validation of endogenous controls for gene expression analysis in microdissected human renal biopsies. Kidney International.

[B13] Frost P, Nilsen F (2003). Validation of reference genes for transcription profiling in the salmon louse, *Lepeophtheirus salmonis*, by quantitative real-time PCR. Veterinary Parasitology.

[B14] Ingerslev HC, Pettersen EF, Jakobsen RA, Petersen CB, Wergeland HI (2005). Expression profiling and validation of reference gene candidates in immune relevant tissues and cells from Atlantic salmon (*Salmo salar *L.). Molecular Immunology.

[B15] Radonic A, Thulke S, Bae H, Muller MA, Siegert W, Nitsche A (2005). Reference gene selection for quantitative real-time PCR analysis in virus infected cells: SARS corona virus, Yellow fever virus, Human Herprevirus-6, Camelpox virus and Cytomegalovirus infections. Virology Journal.

[B16] Olsvik PA, Lie KK, Jordal AO, Nilsen TO, Hordvik I (2005). Evaluation of potential reference genes in real-time RT-PCR studies of Atlantic salmon. BMC Molecular Biology.

[B17] Radonic A, Thulke S, Mackay IM, Landt O, Siegert W, Nitsche A (2004). Guideline to reference gene selection for quantitative real-time PCR. Biochemical and Biophysical Research Communications.

[B18] Garcia-Crespo D, Juste RA, Hurtado A (2005). Selection of ovine housekeeping genes for normalization by real-time PCR; analysis of PrP gene expression and genetic susceptibility to scrapie. BMC Veterinary Research.

[B19] Nishimura M, Koeda A, Suzuki E, Shimizu T, Kawano Y, Nakayama M, Satoh T (2006). Effects of protypical drug-metabolizing enzyme inducers on mRNA expression of housekeeping genes in primary cultures of human and rat hepatocytes. Biochemical and Biophysical Research Communications.

[B20] Gorzelniak K, Janke J, Engeli S, Sharma AM (2001). Validation of endogenous controls for gene expression studies in human adipocytes and preadipocytes. Hormone and Metabolism Research.

[B21] Rey JM, Pujol P, Callier P, Cavailles V, Freiss G, Maudelonde T, Brouillet JP (2000). Semiquantitative reverse transcription-polymerase chain reaction to evaluate the expression patterns of genes involved in the oestrogen pathway. Journal of Molecular Endocrinology.

[B22] de Kok JB, Roelofs RW, Giesendorf BA, Pennings JL, Waas ET, Feuth T, Swinkels DW, Span PN (2005). Normalization of gene expression measurements in tumor tissues: comparison of 13 endogenous control genes. Laboratory Investigation.

[B23] Chen J, Rider DA, Ruan RS (2006). Identification of valid housekeeping genes and antioxidant enzyme gene expression change in the aging rat liver. J Gerontol A Biol Sci Med Sci.

[B24] Celius T, Matthews JB, Giesy JP, Zacharewski TR (2000). Quantification of rainbow trout (*Oncorhynchus mykiss*) zona radiata and vitellogenin mRNA levels using real-time PCR after in vivo treatment with estradiol-17 beta or alpha-zearalenol. Journal of Steroid Biochemistry and Molecular Biology.

[B25] Andreassen TK, Skjoedt K, Korsgaard B (2005). Upregulation of estrogen receptor alpha and vitellogenin in eelpout (*Zoarces viviparys*) by waterborne exposure to 4-tert-octylphenol and 17 beta-estradiol. Comp Biochem Physiol C Toxicol Pharmacol.

[B26] Lee C, Na JG, Lee KC, Park K (2002). Choriogenin mRNA induction in male medaka, *Oryzias latipes *as a biomarker of endocrine disruption. Aquatic Toxicology.

[B27] Hook SE, Skillman AD, Small JA, Schultz IR (2006). Dose-response relationships in gene expression profiles in rainbow trout, *Onchorhynchus mykiss*, exposed to ethinylestradiol. Marine Environmental Research.

[B28] Hook SE, Skillman AD, Small JA, Schultz IR (2006). Gene expression patterns in rainbow trout, *Oncorhynchus mykiss*, exposed to a suite of model toxicants. Aquatic Toxicology.

[B29] Brown M, Robinson C, Davies IM, Moffat CR, Redshaw J, Craft JA (2004). Temporal changes in gene expression in the liver of male plaice (*Pleuronectes platessa*) in response to exposure to ethynyl oestradiol analysed by macroarray and real-time PCR. Mutat Res.

[B30] Knoebl I, Hemmer MJ, Denslow ND (2004). Induction of zona radiata and vitellogenin genes in estradiol and nonylphenol exposed male sheepshead minnows (*Cyprinodon variegates*). Marine Environmental Research.

[B31] Sabo-Attwood T, Kroll KJ, Denslow ND (2004). Differential expression of largemouth bass (*Micropterus salmoides*) estrogen receptor isotypes alpha, beta, and gamma by estradiol. Molecular and Cellular Endocrinology.

[B32] Islinger M, Yuan H, Voelkl A, Braunbeck T (2002). Measurement of vitellogenin gene expression by RT-PCR as a tool to identify endocrine disruption in Japanese medaka (*Oryzias latipes*). Biomarkers.

[B33] Larkin P, Sabo-Attwood T, Kelso J, Denslow ND (2002). Gene expression analysis of largemouth bass exposed to estradiol, nonylphenol, and p,p'-DDE. Biochemistry and Physiology B.

[B34] Bocca C, Gabriel L, Miglietta A (2001). Cytoskeleton-interacting activity of geiparvarin, diethylstilbestrol and conjugates. Chemico-Biological Interactions.

[B35] Chaudoreille MM, Peyrot V, Braguer D, Codaccioni F, Crevat A (1991). Qualitative study of the interaction mechanism of estrogenic drugs with tubulin. Biochemical Pharmacology.

[B36] Sato Y, Sakakibara Y, Oda T, Aizuyokota E, Ichinoseki K (1992). Effect of estradiol and ethinylestradiol on microtubule distribution in Chinese hamster V79 cells. Chem Pharm Bull (Tokyo).

[B37] Hoffmann JL, Torontali SP, Thomason RG, Lee DM, Brill JL, Price BB, Carr GJ, Versteeg DJ (2006). Hepatic gene expression profiling using Genechips in zebrafish exposed to 17α-ethynylestradiol. Aquatic Toxicology.

[B38] Yadetie F, Male R (2002). Effects of 4-nonylphenol on gene expression of pituitary hormones in juvenile Atlantic salmon (*Salmo salar*). Aquatic Toxicology.

[B39] Hoyt PR, Doktycz MJ, Beattie KL, Greeley MS (2003). DNA microarrays detect 4-nonylphenol-induced alterations in gene expression during zebrafish early development. Ecotoxicology.

[B40] Zhang J, Massman GA, Mirabile CP, Figueroa JP (1998). Estrogen stimulates gluceraldehyde-3-phosphate dehydrogenase (GAPDH) mRNA expression in the uterus of non pregnant sheep. Journal of the Society of Gynecological Investigation.

[B41] Zou KY, Ing NH (1998). Oestradiol up-regulates oestrogen receptor, cyclophilin, and gluceraldehyde phosphate dehydrogenase mRNA concentrations in endometrium, but down-regulates them in liver. Journal of Steroid Biochemistry and Molecular Biology.

[B42] Ing NH, Zhang Y (2004). Cell-specific expression of estrogen-responsive genes in the uteri of cyclic, early pregnant and ovariectomized ewes. Theriogenology.

[B43] Diel P, Schulz T, Smolnikar K, Strunck E, Vollmer G, Michna H (2000). Ability of xeno- and phytoestrogens to modulate expression of estrogen-sensitive genes in rat uterus: estrogenicity profiles and uterotropic activity. Journal of Steroid Biochemistry and Molecular Biology.

[B44] Winzer K, Van Noorden CJF, Koher A (2002). Glucose-6-phosphate dehydrogenase: the key to sex-related xenobiotics toxicity in hepatocytes of European flounder (*Platichthys flesus *L.). Aquatic Toxicology.

[B45] Korsgaard B, Mommsen TP (1993). Gluconeogenesis in hepatocytes of immature rainbow trout (*Oncorhynchus mykiss*) – control by estradiol. General and Comparative Endocrinology.

[B46] Sunny F, Jacob A, Oommen OV (2002). Sex steroids regulate intermediary metabolism in *Oreochromis mossambicus*. Endocrine Research.

[B47] Sangiao-Alvarellos, Guzman JM, Laiz-Carrion R, Minguez JM, Martin del Rio MP, Mancera JM, Soengas JL (2004). Actions of 17β-estradiol on carbohydrate metabolism in liver, gills and brain of gilthead sea bream *Sparus aurata *during acclimation to different salinities. Marine Biology.

[B48] Peter MCS, Oomen OV (1989). Oxidative metabolism in a teleost, *Anabas testudineus *Bloch: effect of testosterone and estradiol-17β on hepatic enzyme activities. Fish Physiology and Biochemistry.

[B49] Thellin O, Zorzi W, Lakaye B, De Borman B, Coumans B, Hennen G, Grisar T, Igout A, Heinen E (1999). Housekeeping genes as internal standards: use and limits. Journal of Biotechnology.

[B50] Solanas M, Moral R, Escrich E (2001). Unsuitability of using ribosomal RNA as a loading control for Northern blot analysis related to the imbalance between messenger and ribosomal RNA content in rat mammary tumors. Analytical Biochemistry.

[B51] Brunner AM, Yakovlev IA, Strauss SH (2004). Validating internal controls for quantitative plant gene expression studies. BMC Plant Biology.

[B52] Rubie C, Kempf K, Hans J, Su T, Tilton B, Georg T, Brittner B, Ludwig B, Schilling M (2005). Housekeeping gene variability in normal and cancerous colorectal, pancreatic, esophageal, gastric and hepatic tissues. Molecular and Cellular Probes.

[B53] Lattier DL, Gordon DA, Burks DJ, Toth GP (2001). Vitellogenin gene transcription: a relative quantitative exposure indicator of environmental estrogens. Environmental Toxicology and Chemistry.

[B54] Elskus AA (2004). Estradiol and estriol suppress CYP1A expression in rainbow trout primary hepatocytes. Marine Environmental Research.

[B55] Desbrow C, Routledge EJ, Brighty GC, Sumpter JP, Waldock M (1998). Identification of estrogenic chemicals in STW effluent. 1. Chemical fractionation and in vitro biological screening. Environmental Science and Technology.

[B56] Filby AL, Tyler CR (2005). Molecular characterization of estrogen receptors 1, 2a and 2b and their tissue and ontogenic expression profiles in fathead minnow (*Pimephales promelas*). Biology of Reproduction.

[B57] Altschul SF, Gish W, Miller W, Myers EW, Lipman DJ (1990). Basic local alignment search tool. Journal of Molecular Biology.

[B58] Thompson JD, Gibson TJ, Plewniak F, Jeanmougin F, Higgins DG (1997). The ClustalX windows interface: flexible strategies for multiple sequence alignment aided by quality analysis tools. Nucleic Acids Research.

[B59] Livak KJ, Schmittgen TD (2001). Analysis of relative gene expression data using real-time quantitative PCR and the 2^-ΔΔCT ^method. Methods.

[B60] Soong R, Ruschoff J, Tabiti K (2000). Detection of colorectal micrometastasis by quantitative RT-PCR of cytokeratin 20 mRNA. Roche Diagnostics internal publication.

[B61] Pfaffl MW (2001). A new mathematical model for relative quantification in real-time RT-PCR. Nucleic Acids Research.

