# Supplementary material for: Appropriate 'housekeeping' genes for use in expression profiling the effects of environmental estrogens in fish
Source: BMC Mol Biol. 2007 Feb 8;8:10. doi: 10.1186/1471-2199-8-10 (PMC1802086; doi:10.1186/1471-2199-8-10)
Supplement: Additional file 1 — Table of PCR primer sequences, annealing temperatures (Ta), cycle numbers and product sizes used for cloning the candidate 'housekeeping' genes. [file 1471-2199-8-10-S1.pdf]

Additional file 1: Table of PCR primer sequences, annealing temperatures (T_a), cycle numbers and product sizes used for cloning the candidate 'housekeeping' genes.

**Target gene**	**Primer name**	**Type**	**Primer sequence (5'-3')**	**T_a used for PCR (^oC)**	**No. cycles**	**Product size (bp)**
18S rRNA	18S rRNA Forward	'Core PCR'	GCGGCGACGACTCTTTCGAATGTC	54.0	35	768
18S rRNA	18S rRNA Reverse	'Core PCR'	AGTTCCGACCGTAAACGATG			
rpl8	*rpl8* Forward	'Core PCR'	CTCCGTCTTCAAAGCCCATGT	54.0	35	708
rpl8	*rpl8* Reverse	'Core PCR'	TGTTCCTCGCAGTCTGCCAG			
ef1a	*ef1a* Forward 1	'Core PCR 1'	GTGACAACGTTGGCTTCAACG	54.8	25	377
ef1a	*ef1a* Reverse 1	'Core PCR 1'	CCTCATGTCACGCACAGCAAA			
ef1a	*ef1a* Forward 2	'Core PCR 2'	AGGCTGGTATCTCCAAGAACG	54.0	25	564
ef1a	*ef1a* Reverse 2	'Core PCR 2'	CGTTGAAGCCAACGTTGTCAC			
ef1a	*ef1a* 5'-RACE	'5'-RACE'	GGTCTGTCCGTTCTTGGAGATACCAGC	68.0	30	470
ef1a	*ef1a* 3'-RACE	'3'-RACE'	GCAAGAAGCTTGAGGACAACC	55.0	40	494
