# Supplementary material for: Appropriate 'housekeeping' genes for use in expression profiling the effects of environmental estrogens in fish
Source: BMC Mol Biol. 2007 Feb 8;8:10. doi: 10.1186/1471-2199-8-10 (PMC1802086; doi:10.1186/1471-2199-8-10)
Supplement: Additional file 2 — Table of real-time PCR primer sequences, real-time PCR product sizes, annealing temperatures (Ta), and standard curve (mean Ct vs. log cDNA dilution) slopes, PCR efficiencies (E), and correlation coefficients for the candidate 'housekeeping' genes. [file 1471-2199-8-10-S2.pdf]

Additional file 2: Table of real-time PCR primer sequences, real-time PCR product sizes, annealing temperatures (T_a), and standard curve (mean C_t vs. log cDNA dilution) slopes, PCR efficiencies (*E*), and correlation coefficients for the candidate 'housekeeping' genes.

**Target gene**	**Sense primer (5'-3')**	**Antisense primer (5'-3')**	**Product (bp)**	**T_a (^oC)**	**Slope**	***E***	**R_2**
18S rRNA	AATGTCTGCCCTATCAACTTTC	TGGATGTGGTAGCCGTTTC	134	59.0	-3.055	2.12	0.998
rpl8	CTCCGTCTTCAAAGCCCATGT	TCCTTCACGATCCCCTTGATG	162	60.0	-3.015	2.14	0.999
ef1a	AAGAGCGTTGAGAAGAAAG	GAGTGCCCAGGTTTAGAG	154	55.0	-3.229	2.04	0.999
g6pd	ACTCAACTGTCCCTAAAG	CCTCTGCCTTCCTCTC	132	52.5	-3.005	2.15	0.996
bactin	GAATCCCAAAGCCAACAG	AACACCATCACCAGAGTC	148	59.0	-2.794	2.28	0.994
gapdh	CAGACGCTTCCCACAAAC	TCACCAGACGCCCAATG	91	58.0	-3.533	1.92	0.999
hprt1	GATGAAGAGCAAGGTTATGAC	ACACAGAGCAACGATATGG	165	58.0	-2.804	2.27	0.992
tbp	CTCAAGGGCTGGCTTCTC	ACTGGCTGTGGTGTAAGAC	97	59.0	-2.953	2.18	0.998
